# Threatened Abortion

**Published:** 1899-01

**Authors:** John S. Moreman

**Affiliations:** Louisville, Ky.; Obstetrician to St. Ann’s Maternity Hospital


### THREATENED ABORTION.

By JOHN S. MOREMAN, M.D., LOUISVILLE, KY.

Obstetrician to St. Ann's Maternity Hospital.

A very common accident to pregnant women is the occurrence of abortion. The causes which lead to the production of abortion are very plentiful and comprehend nervous excitement and injuries and accidents of various kinds. It is of the utmost advantage to the patient to prevent the occurrence of abortion and thus save the life of the unborn, and also protect the patient against dangers incident upon abortion. We are not fully abreast with the times if we underestimate the importance of abortion as an indirect as well as a direct cause of a large amount of mortality. Abortion is produced by professionals and from their work we are constantly called to see a large number of cases of peritonitis from this cause. Of course the limits of this paper have nothing to do with criminal abortion in any way and reference to it above was only for the purpose of showing a dire result traceable to it, that will also be seen resulting from abortion when due to causes far removed from that of a criminal intention. When we are called to see a patient who is pregnant and has regular pains in the region of the uterus, whether there is hemorrhage or not, we must lose no time in bringing about a cessation of these pains. When there has been hemorrhage, we must not be discouraged, since Playfair and other good obstetrical observers testify that even after this has been considerable, we shall often be successful in quieting the pains of the patient, and the patient will go on to a full term. Not common is it to see these patients get along well after the hemorrhage has been considerable.

When hemorrhage is small and the uterus is not dilated we may, by instituting prompt and effective treatment, bring the patient around to a safe point. It is of the first importance for the obstetrician to make a vaginal examination to ascertain the exact conditions that exist in every case. If upon examination the dilation is found to be extreme, and there is no reasonable hope of the patient being spared of abortion, then measures should be instituted that will speedily deliver the patient, and in this allow her to escape the danger of probable excessive hemorrhages and probable death. The first thing to be done toward the prevention of abortion is to secure for the patient absolute rest and quiet. The assumption of the recumbent position is an absolute necessity in all cases. The patient should undress and occupy a comfortable position in the bed. In cases where pains are produced by excitement or fright, this will often be sufficient to bring about a cessation of the pains, but this alone can not be depended upon, and the writer does not desire to recommend it. It is necessary to give an agent which acts not only as an uterine sedative but also as a quieting agent to the general nervous system. When we speak of the necessity of rest, it should be borne in mind by the obstetrician that the patient must be comfortable in the bed. She must not have too many bed quilts put over her, for these will prove burdensome and aggravating. The patient must not be allowed to get up on the chamber to go to stool or empty her bladder. These acts frequently undo all that we have accomplished up to this time. In a word, no exercise must be allowed. The bed pan will prevent much exertion that would otherwise be necessary in these cases. The diet taken by these patients must be very light and never should the patient be allowed to eat very stimulating food or fill her stomach with food which is hard to digest. When the patient is first seen and the pains are coming with regularity, the administration of a hypodermic injection of morphine may be made with advantage. When, however, the patient is very susceptible to pain, as some extremely nervous women are, this is to be avoided as it will very often so excite the patient that she will be made worse. To give it by the mouth it should be given with five grains of oxalate of cerium to overcome the nauseating tendency of morphine. No more than one dose of morphine should be given except in extraordinary cases. After the first dose, the best remedy that can be given to prevent a further continuance of these pains is aletris cordial. It often happens that we will get relief in a very short period of time. Not infrequently does it happen that relief follows a third or fourth dose and attack is entirely brought to a successful termination. There should be given no opium to any patient if that can be avoided. Urgency of pains only demands it, but when the pains are not intense, reliance should be placed upon putting the patient to bed and giving aletris cordial.

Aletris cordial was first brought to my notice on account of its value in amenorrhœa and dysmenorrhœa, but I find for it a wider sphere of usefulness.—*New England Medical Monthly.*

---

ASTRONOMICAL.

O brilliant were the stars above!
A dazzling simulacrum;
She slipped on a banana peel,
And lit upon her sacrum!

